# Civic Engagement in Retirement and the Socioemotional Experience of Pandemic Time

**DOI:** 10.1093/geroni/igab046.2589

**Published:** 2021-12-17

**Authors:** Boroka Bo

**Affiliations:** University of California, Berkeley, Berkeley, California, United States

## Abstract

This research integrates literature from the sociology of the life course, sociology of emotions and the sociology of time to examine how Socioeconomic Status (SES) influenced retiree civic engagement during the COVID-19 pandemic. I find that SES framed both the social experience of time and the prevalent emotions experienced by retirees while physically distancing during the early days of the pandemic. These individual-level experiences translated to markedly different blueprints for civic engagement. High-SES retirees were more likely to ‘go global’, organizing to advocate for their interests. Conversely, low-SES retirees were more likely to ‘turn in’, minimizing their civic engagement. My findings reveal how existing sociopolitical inequalities may become further entrenched in public health crises. Policies aimed at combating inequalities in later life also need to consider socioemotional and sociotemporal factors.

